# Gene Therapy Regenerates Protein Expression in Cone Photoreceptors in *Rpe65^R91W/R91W^* Mice

**DOI:** 10.1371/journal.pone.0016588

**Published:** 2011-02-03

**Authors:** Corinne Kostic, Sylvain Vincent Crippa, Vérène Pignat, Alexis-Pierre Bemelmans, Marijana Samardzija, Christian Grimm, Andreas Wenzel, Yvan Arsenijevic

**Affiliations:** 1 Unit of Gene Therapy and Stem Cell Biology, Jules-Gonin Eye Hospital, University of Lausanne, Lausanne, Switzerland; 2 Laboratory for Retinal Cell Biology, Department of Ophthalmology, University of Zürich, Zürich, Switzerland; Innsbruck Medical University, Austria

## Abstract

Cone photoreceptors mediate visual acuity under daylight conditions, so loss of cone-mediated central vision of course dramatically affects the quality of life of patients suffering from retinal degeneration. Therefore, promoting cone survival has become the goal of many ocular therapies and defining the stage of degeneration that still allows cell rescue is of prime importance. Using the *Rpe65^R91W/R91W^* mouse, which carries a mutation in the Rpe65 gene leading to progressive photoreceptor degeneration in both patients and mice, we defined stages of retinal degeneration that still allow cone rescue. We evaluated the therapeutic window within which cones can be rescued, using a subretinal injection of a lentiviral vector driving expression of RPE65 in the *Rpe65^R91W/R91W^* mice. Surprisingly, when applied to adult mice (1 month) this treatment not only stalls or slows cone degeneration but, actually, induces cone-specific protein expression that was previously absent. Before the intervention only part of the cones (40% of the number found in wild-type animals) in the *Rpe65^R91W/R91W^* mice expressed cone transducin (GNAT2); this fraction increased to 64% after treatment. Correct S-opsin localization is also recovered in the transduced region. In consequence these results represent an extended therapeutic window compared to the *Rpe65^-/-^* mice, implying that patients suffering from missense mutations might also benefit from a prolonged therapeutic window. Moreover, cones are not only rescued during the course of the degeneration, but can actually recover their initial status, meaning that a proportion of altered cones in chromophore deficiency-related disease can be rehabilitated even though they are severely affected.

## Introduction

Two main factors compose the success of a therapy: the efficiency of the therapeutic strategy and the status of the cells to save. As good as the treatment can be, it will never offer its full action if it is applied too late in the natural course of the disease. Thus the detailed description of the disease progression is of prime importance to determine the latest stage that can be targeted for success of the treatment before irreversible damage. Extensive technological advances in therapies and imaging now offer a large panel of possibilities to heal ophthalmologic diseases that were so far incurable.

Gene therapy has found in ophthalmology the advantage of a field that includes many different monogenic hereditary diseases of a relatively accessible organ, offering scientists a rich ground to develop different strategies of gene therapy. Confirming the dynamism in this field, RPE65 gene replacement for Leber congenital amaurosis (LCA) [1] is now in Phase I/II clinical trials driven by three different teams all using AAV2/2 vectors [2]–[5]. Most gene transfer studies show success only when the treatment was applied early or even before the onset of the degeneration. In consequence, it is still unclear whether cones can recover a normal state when treatment occurs during advanced stages of cone degeneration. In many models of retinal degeneration, rods die first, and the following lack of trophic support as well as the alteration in the architectural structure, induces cone death. Thus cone rescue and rehabilitation can not be evaluated in such models because cone survival will inevitably be impaired in these affected retinas. From this point of view, Rpe65-deficient mice are interesting models to study cone degeneration, since in this case cone loss occurs before rod loss [6]. RPE65 is expressed in the retinal pigment epithelium [7], which is juxtaposed to the neuroretina. This protein belongs to the visual cycle, and is responsible for the regeneration of 11-*cis*-retinal, the chromophore essential for the visual pigments (contained in rod and cone photoreceptors) to capture light. Deletion of the RPE65 isomerase blocks the generation of this retinoid resulting in a severe loss of photoreceptor function as well as cone and rod death [7]. Conversely, the restoration of RPE65 expression allows to rescue rod function and the proof of principle of this approach was widely demonstrated in mouse [8]–[14] and dog models [15]–[17]. Cone function can also be rescued after RPE65 gene transfer, but the therapeutic window is very limited in RPE65 knockout mice which undergo a rapid cone degeneration [13], [18]. The first results of the Phase I clinical trials, started end of 2007, have shown no adverse effects so far and even improvement of some visual functions in certain cases [2]–[5], [19]. These encouraging results lead to the second step of these trials consisting in treating younger patients.

As in most progressive diseases, the time of intervention during the disease progression determines the efficiency of the rescue [10]. Measurements of the retinal thickness in young RPE65-affected patients revealed inter-individual differences not clearly related to age [20], which might have a consequence on the efficiency of a potential treatment. The general genetic background as well as modifier genes influencing RPE65 function may be important factors in the phenotypic variability [21]. Moreover, among the patients suffering from RPE65 gene defects, more than half bear missense mutations [22]. These mutations are extremely heterogeneous, thus residual enzyme activity can be expected in certain cases which possibly explains the high variability of the disease severity [23]–[26]. The R91W mutation was described in patients suffering from an early-onset retinal degeneration [25], who experience useful cone-mediated vision in the first decade of life [26]. As suggested by this favorable condition in young patients, a low level of the mutated protein is present and allows production of small amounts of 11-*cis*-retinal in the *Rpe65^R91W/R91W^* mouse model [27], where cone degeneration is observed but with slower kinetics compared to the null background for RPE65 [28].

Indeed, in *Rpe65* ^-/-^ mice, cones rapidly degenerate in young adults, resulting in an almost complete loss of cones at 8 weeks of age [6]. Expression of cone transducin α-subunit (GNAT2) as well as of both cone opsins (short- (S-) and middle-/long- (M/L-) wavelength opsins) is already severely impaired at 1 month of age [6]. Rods degenerate more slowly, with residual function demonstrated up to 24 months [29]. Consistent with these observations, we previously showed that, using lentiviral-mediated RPE65 gene transfer, cone rescue in *Rpe65* ^-/-^ mice is limited to injection at P5 and absolutely inefficient if the treatment occurs at 1 month [13]. The cones appear to be too severely affected at 1 month of age in *Rpe65^-/-^* mice to benefit from the revitalization of the visual cycle. The reason for cone death is not yet fully determined however there are now several reports suggesting that the lack of 11-*cis*-retinal is the main cause for cone degeneration in different mouse models (*Lrat^-/-^* [30], [31], *Irbp^-/-^* [32]). Because in *Rpe65* ^-/-^ mice the level of 11-*cis*-retinal is undetectable, cone opsin mislocalization becomes evident early in life [28], [33] and the cones rapidly degenerate. Treatment with 9-*cis*-retinal [32], [33] or gene transfer of RPE65 [13] corrects the localization of the cone opsins to the outer segments, thus demonstrating the important role of 11-*cis*-retinal for the accurate trafficking of the cone opsins and cone survival. In RPE65-deficient patients, cone loss is observed in both the fovea [34] and the periphery, which is mainly populated by S-cones and first affected [35], [36]. We thus took advantage of the milder cone phenotype of the *Rpe65^R91W/R91W^* mouse model to assess the time-frame limit for effective cone rescue and whether gene therapy preserves only the remaining healthy cones or can revitalize severely affected cones. Such knowledge should bring broad applications to therapies aiming to rescue cones during the course of their degeneration.

## Results

### Early LV-RPE65 treatment in *Rpe65^R91W/R91W^* mice improves retinal function

A gene replacement strategy requires expression of the therapeutic gene in the appropriate cell type. In order to target the retinal pigment epithelium (RPE), we used a HIV-1 derived lentiviral vector known to target RPE cells very efficiently [37], [38]. Additionally, to restrict expression of the transgene in RPE, we used a 0.8 kb fragment of the human RPE65 promoter (R0.8) [13], [39], [40]. We thus restored expression of wild type RPE65 in *Rpe65^R91W/R91W^* mice by intraocular injection in postnatal mice (postnatal day 5, (P5), Fig S1A) or by subretinal injection in adult mice of LV-RPE65 vector (Fig S1C). This vector drives specific expression of the RPE65 protein in RPE although in rare cases the LV-GFP control vector also drives expression in Müller cells as described previously (Fig S1B, [13]). We can distinguish the RPE65 transgene expression from the endogenous mutant protein mostly because of an increased RPE65 immunostaining within the RPE near the injection site (Fig S1C, arrow). The area of transduction obtained was similar for injection both in P5 and in 1 month-old animals, ranging from 0.1 to 3 mm^2^ (Fig S1D, this heterogeneity in the transduction area is due to the injection procedure). The eyes with the wider zone of transduction have thus around 20% of the retina expressing the wild type RPE65 protein.

**Figure 1:**
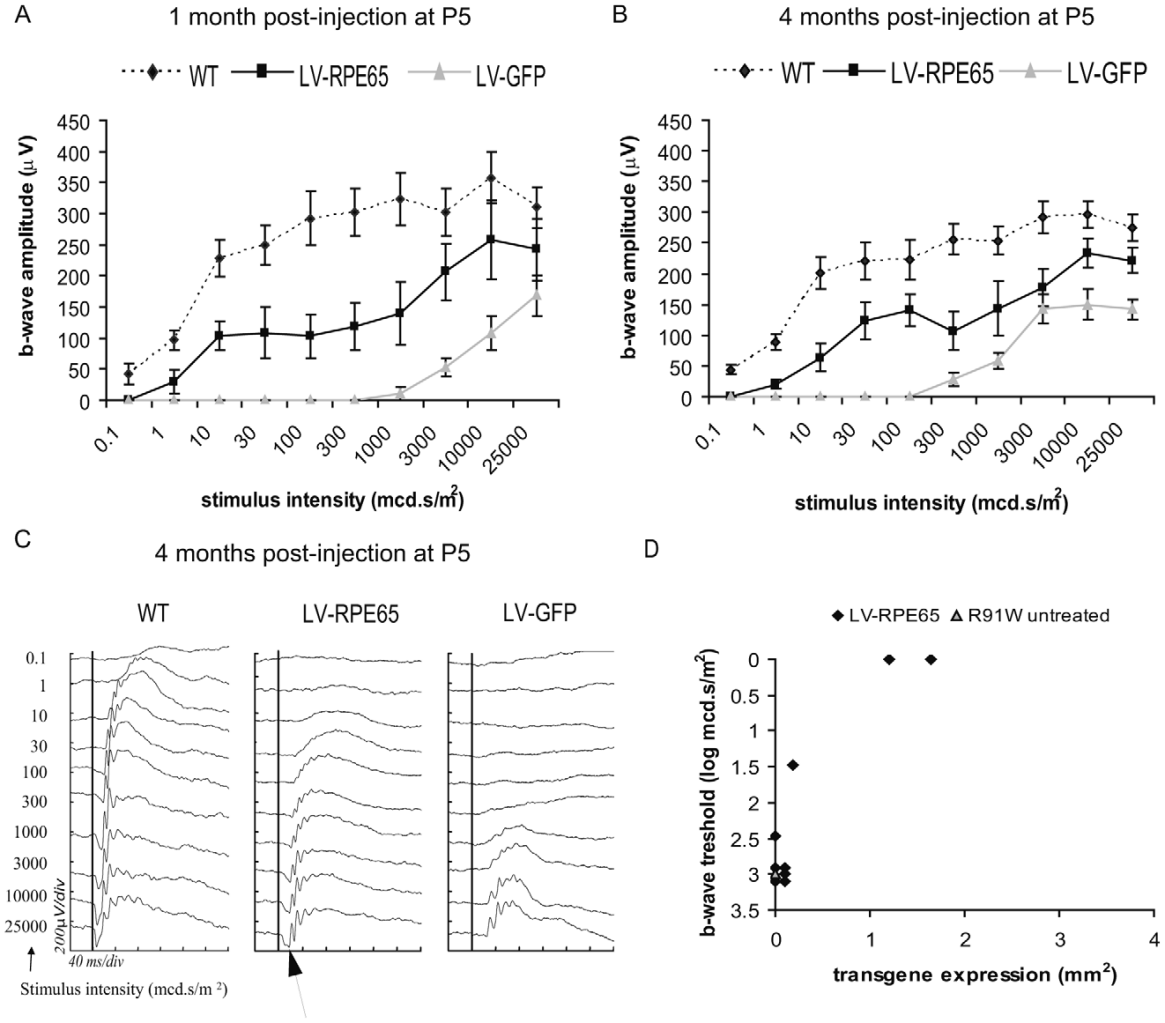
Lentiviral-mediated *Rpe65* gene transfer at P5 in *Rpe65^R91W/R91W^* mice improved retinal sensitivity in scotopic conditions. (A,B) Graphs representing the b-wave amplitudes of scotopic ERGs versus the intensity of the stimuli 1 month (A) and 4 months post injection (B) of *Rpe65^R91W/R91W^* mice treated at P5 showed an improved sensitivity of the response in the group of animals treated with LV-RPE65 (squares) compared to the group treated with LV-GFP (triangles). The response of the LV-RPE65 group was determined by the average response of eyes showing a positive RPE65 labeling (n = 3). Statistical analysis using an ANOVA for repeated measurements showed significant group, stimuli intensity and group versus stimuli intensity effects, p<0.01. For comparison, the scotopic response of age-matched wt animals (SV129) is shown (diamonds). Data are represented as mean ± standard error of the mean (SEM). (C) Representative ERG tracings of age-matched WT, LV-RPE65-treated and LV-GFP-treated eyes 4 months post injection at P5 illustrate the improvement of the scotopic response of LV-RPE65-treated eyes. Note the increase in the a-wave as well (arrow). The black vertical lines crossing the ERG tracings represent the stimulus time. (D) Plotting the b-wave threshold, corresponding to the lowest stimulus which induces an ERG response, to the transgene expression area shows that the improved b-wave threshold correlates with RPE65 expression. LV-RPE65-treated animals (diamonds) without noticeable transgene expression present a threshold similar to the average of age-matched untreated animals (triangle).

In order to evaluate the efficiency of RPE65 gene transfer to restore a retinal function, the electrical activity of the retina was recorded by electroretinography (ERG) 1 month and 4 months post injection at P5. A control group injected with a vector containing a GFP reporter gene (LV-GFP) was examined in parallel. As previously described [27], *Rpe65^R91W/R91W^* retinal function in photopic conditions is recordable. The photopic responses of *Rpe65^R91W/R91W^* mice showed no differences in sensitivity, only a diminished b-wave amplitude with age compared to wild type mice. This particular response is hypothesized to be a mixed cone-rod response and renders the impact of gene transfer on cone function difficult to assess. Consistently, there was no difference in the photopic responses between the LV-RPE65 group, the LV-GFP group, untreated animals or wild type animals (data not shown). However, *Rpe65^R91W/R91W^* mice have reduced sensitivity to the rod response which can be measured in scotopic conditions with low intensity stimuli. In LV-RPE65-treated eyes expressing RPE65, a 3-log increase in the b-wave response threshold in the scotopic ERG clearly showed that the rod system acquires an improved sensitivity (Fig 1A). This improvement of scotopic sensitivity was maintained over 3 more months corresponding to the end of the experiment (Fig 1B) and the maximum b-wave amplitude was 80% of wild-type. Moreover, the b-wave shape at higher stimuli was extended compared to LV-GFP or untreated animals, which is characteristic of the cone input for these light intensities. Notably, at 4 months of age, the a-wave (reflecting the photoreceptor activity, Fig 1C, arrow) became evident on the ERG tracing of LV-RPE65-treated eyes whereas it was undetectable for LV-GFP-treated eyes (Fig 1C). The positive effect of LV-RPE65 injection on rod sensitivity was clearly correlated with the efficiency of gene transfer (Fig 1D). The limited number of eyes expressing RPE65 (Fig 1D) is mainly due to the difficulty of the injection in P5 pups as we already noticed in our previous study [13]. Indeed, in our hands, this type of injection has a 30% success rate.

We previously showed restoration of cone function after RPE65 gene transfer using *Rpe65^-/-^ Gnat1^-/-^* mice [13]. In order to demonstrate cone functional rescue after RPE65-gene transfer in *Rpe65^R91W/R91W^* mice as well, we attempted to use the same strategy using lentiviral vector injection in *Rpe65^R91W/R91W^* mice with a rod deficiency background. As we demonstrated that rods compete cones for 11-cis supply in the *Rpe65^R91W/R91W^* mice [28] and might thus decrease the efficiency of gene transfer-mediated cone rescue, we undertook lentiviral treatment in *Rpe65^R91W/R91W^ Rho^-/-^* mice which should minimize this phenomenon thanks to the absence of the rhodopsin protein. However, so far, our P5 injections could not restore a convincing photopic response as measured by ERGs 3 weeks post injection (data not shown).

### Early LV-RPE65 treatment in *Rpe65^R91W/R91W^* mice protects cones

**Figure 2:**
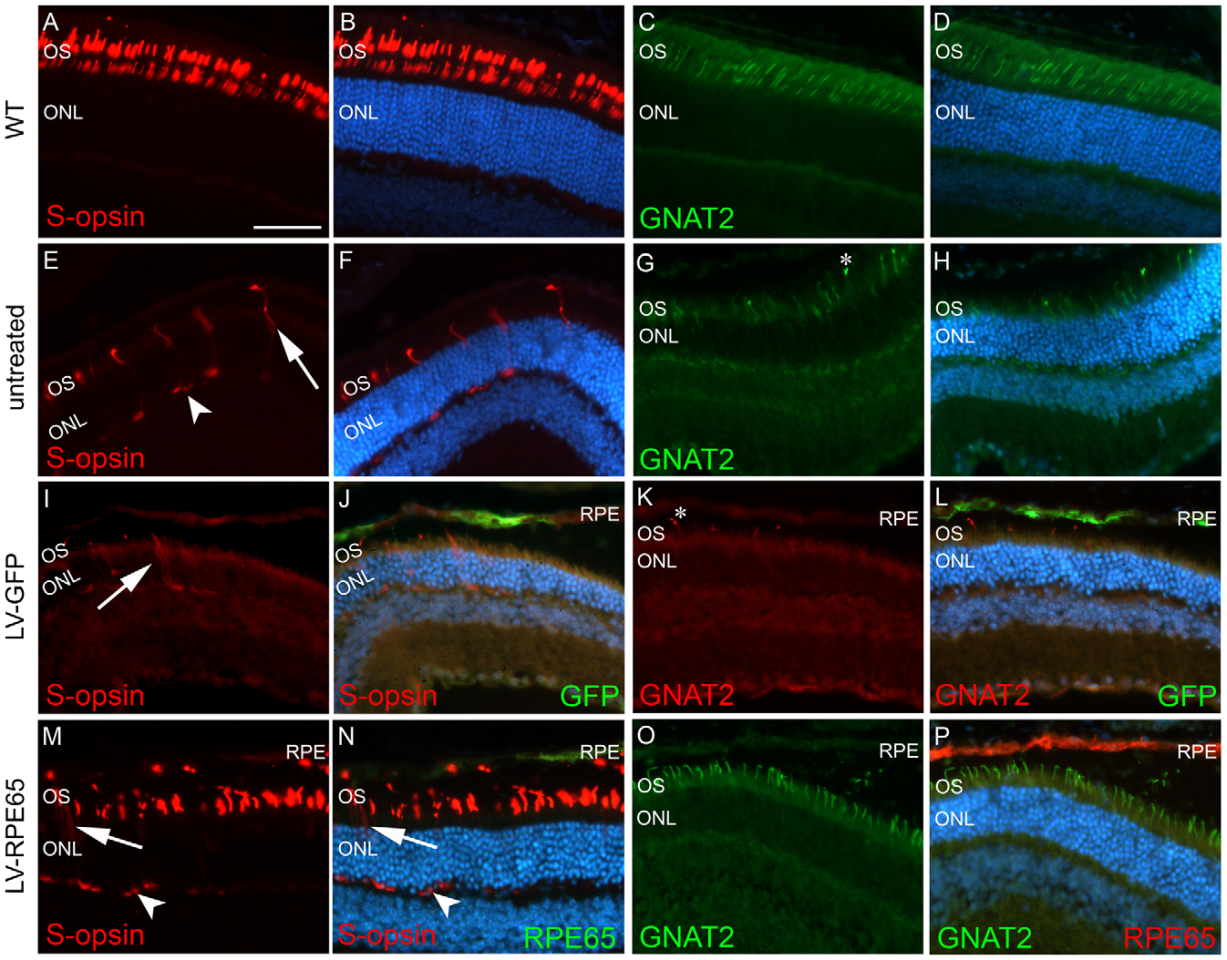
LV-RPE65 injected at P5 protects cones up to 4 months post injection. (A–D) In wild type mice, S-opsin (red) and GNAT2 (green) expressions are localized to outer segments. (E,F) In 4-month-old untreated *Rpe65^R91W/R91W^* mice, S-opsin is highly mislocalized in the cone photoreceptors and the labeling shows distinctly cell bodies (in red, arrow) and feet (in red, arrowhead) of cones. (G,H) At the same age, the cone transducin labeling (GNAT2 in green) is strongly reduced with only minor signals in some tips of shortened outer segments (star). (I,J) Similarly to untreated animals, LV-GFP-injected *Rpe65^R91W/R91W^* mice show reduced and mislocalized expression of S-opsin (red, arrow), even in the region of GFP expression (green). (K,L) The strong reduction of GNAT2 labeling (red, star) is also evident in the LV-GFP-treated region (GFP in green). (M,N) On the contrary, after LV-RPE65 injection at P5, in the region of RPE65 expression (green), strong S-opsin expression is observed in cone outer segments (red) while in the region devoid of WT RPE65 expression there is evidence of S-opsin mislocalization to the cell body (arrow) and synaptic termini (arrowhead). (O,P) RPE65 gene transfer also rescues GNAT2 expression (green) in cone outer segments in the region of RPE65 expression (red). GNAT2: cone-specific transducin α-subunit; S-opsin: short wavelength cone opsin; the scale bar indicated in A represents 50 µm for A-P.

**Figure 3:**
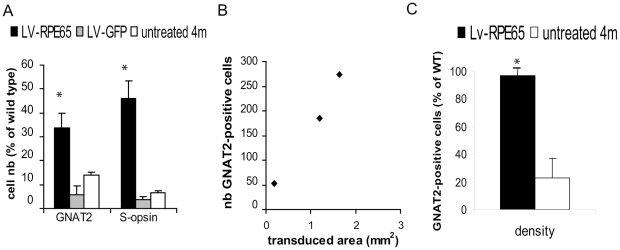
LV-RPE65 treatment of *Rpe65^R91W/R91W^* mice at P5 restores cone density comparable to wild type. (A) Quantification of the number of outer segments expressing GNAT2 or S-opsin proteins in sections positioned in the middle of the transduced area was compared between LV-RPE65-treated, LV-GFP-treated and untreated animals. The LV-RPE65 group shows a clear improvement in the number of cells correctly expressing these cone markers (p<0.01, stars). Data are represented as mean ± standard error of the mean (SEM). (B) When we plot the transduced area to the number of GNAT2-labeled outer segments, a clear correlation shows that the wider the transgene expression area, the more GNAT2-positive outer segments. (C) Finally, we estimated the density of cones correctly expressing the GNAT2 in the outer segments in the middle of the transduced area of LV-RPE65-treated eyes. When compared to the density of cones in wild-type animals at the same position, we observed that LV-RPE65 injection at P5 allows to rescue 100% of the cones (p<0.001, star). Data are represented as mean ± SEM. 4m: 4 month old.

To examine the extent of cone rescue after *Rpe65* gene transfer at P5, we performed immuno-histochemical analysis at the final point of the experiment of 4 months post injection. In untreated *Rpe65^R91W/R91W^* mice of 4 months of age, the different cone markers S-opsin (Fig 2E,F, [28]), M/L-opsin [28] and GNAT2 were strongly reduced (Fig 2G,H, [28]) compared to wild type animals (Fig 2A–D). As described in the literature, deprivation of 11-*cis* retinal affects S-opsin cellular localization and the remaining cones expressing this protein at this age presented a mislocalized labeling all along the cell body (Fig 2E, arrow) and at synaptic endings (Fig 2E, arrowhead). Similar patterns of expression were observed in LV-GFP-treated animals (Fig 2I–L). However, in the region of wt *Rpe65* gene transfer, a clear expression and correct localization of these markers in the cone outer segments was demonstrated (Fig 2M–P). Quantification of the number of cells positive for these markers in the central section of the transduced area of three eyes with the largest transduction area revealed statistically significant differences between the LV-RPE65 group (S-opsin: 46±6% of wild type, GNAT2: 34±6% of wild type) and the LV-GFP (S-opsin: 6±4% of wild type, GNAT2: 4±1% of wild type) or untreated groups (S-opsin: 14±1% of wild type, GNAT2: 7±1% of wild type) (p<0.01, Fig 3A). The improvement in the S-opsin labeling was possible because the transduced region was central or even ventral in those eyes. However, a fourth eye with a limited transduction in the dorsal region showed little increase in the S-opsin cells because normal S-opsin expression is minimal in this region. Moreover, the number of GNAT2 (Fig 3B) and S-opsin (data not shown) labeled cells positively correlated to the size of transduced area.

Furthermore, we determined the density of cones in the center of the transduced area for 3 eyes using the GNAT2 marker which is expressed in both S- and M/L-cones. For each treated region, we expressed the cone density as a percentage of wild type cone densities, determined at the same eye position in an average of 3 to 4 wt control eyes. Thus we can reliably evaluate the efficiency of cone rescue for each eye with regards to the location of the transduced region. This method allowed to assess efficiency of the rescue at the site of wt gene expression and to avoid underestimation of this effect because of the limited transduced area (in the best case 20% of the retina). This quantification shows that LV-RPE65 totally rescues the pattern of GNAT2 expression with a density identical to wild type while untreated mice (determined at the same location) presented only 23% of the wild type GNAT2-cone density (Fig 3C).

### LV-RPE65 treatment in adult *Rpe65^R91W/R91W^* mice improves retinal function

**Figure 4:**
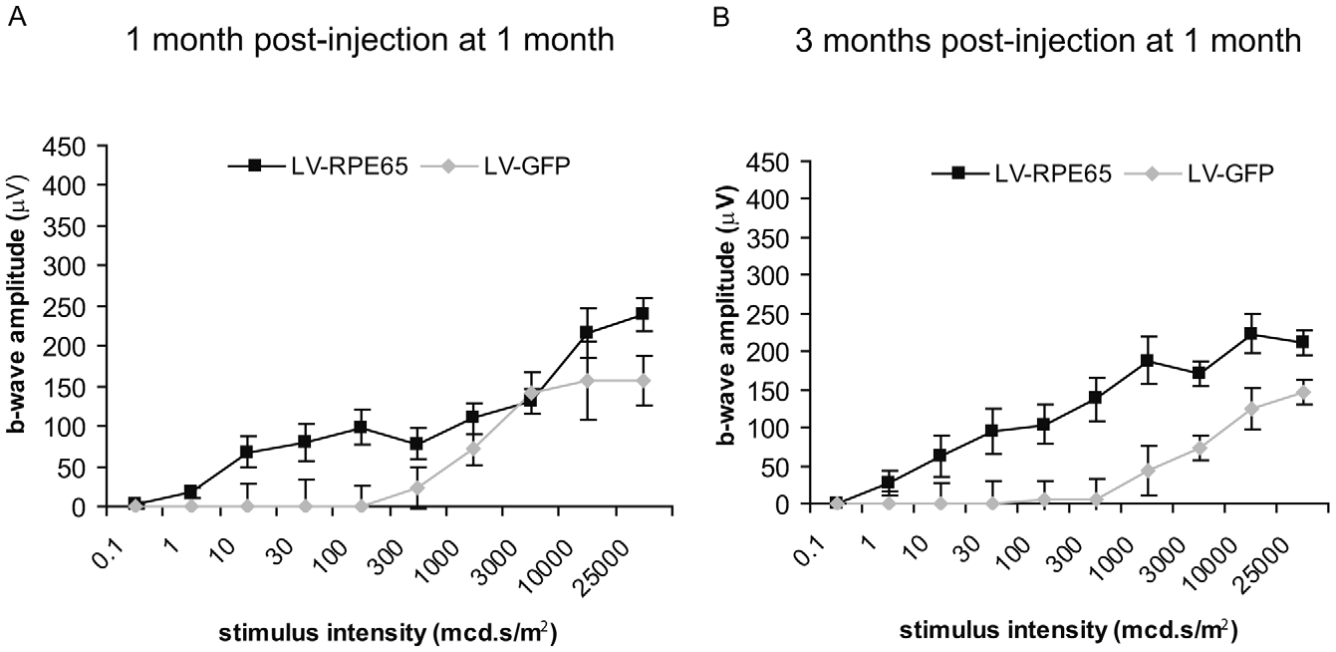
LV-RPE65 injection in 1 month-old *Rpe65^R91W/R91W^* mice improves retinal function. (A,B) Retinal function was assessed by ERG in dark-adapted conditions in *Rpe65^R91W/R91W^* mice 1 month (A) and 3 months (B) post injection at 1 month of age of either LV-RPE65 or LV-GFP. Improved sensitivity was noticed for the LV-RPE65 group compared to the LV-GFP group. ANOVA for repeated measures showed for both time points significant group, stimuli intensity and group versus stimuli intensity effects, p<0.01. Data are represented as mean ± SEM.

We previously showed that *Rpe65* gene transfer at post natal day 5 in the *Rpe65* knockout model allows to protect cone survival and function [13]. In contrast, the treatment of *Rpe65^-/-^* at 1 month of age does not allow cone rescue despite a good expression of the therapeutic *Rpe65* gene in RPE [13]. Our present study aimed to determine whether in *Rpe65^R91W/R91W^* mice, which show a milder cone degeneration [27], intervention at a later stage still allows preservation of cone photoreceptors. We performed subretinal injections of LV-RPE65 in 1 month-old *Rpe65^R91W/R91W^* mice. The retinal electrical activity was recorded 1 and 3 months post-injection by ERG. As observed with the treatment at P5, LV-RPE65-treated eyes displayed a clear increase in the sensitivity of the scotopic b-wave 1 month post injection (Fig 4A). This effect was sustained to the end point of the analysis 3 months post injection (Fig 4B).

**Figure 5:**
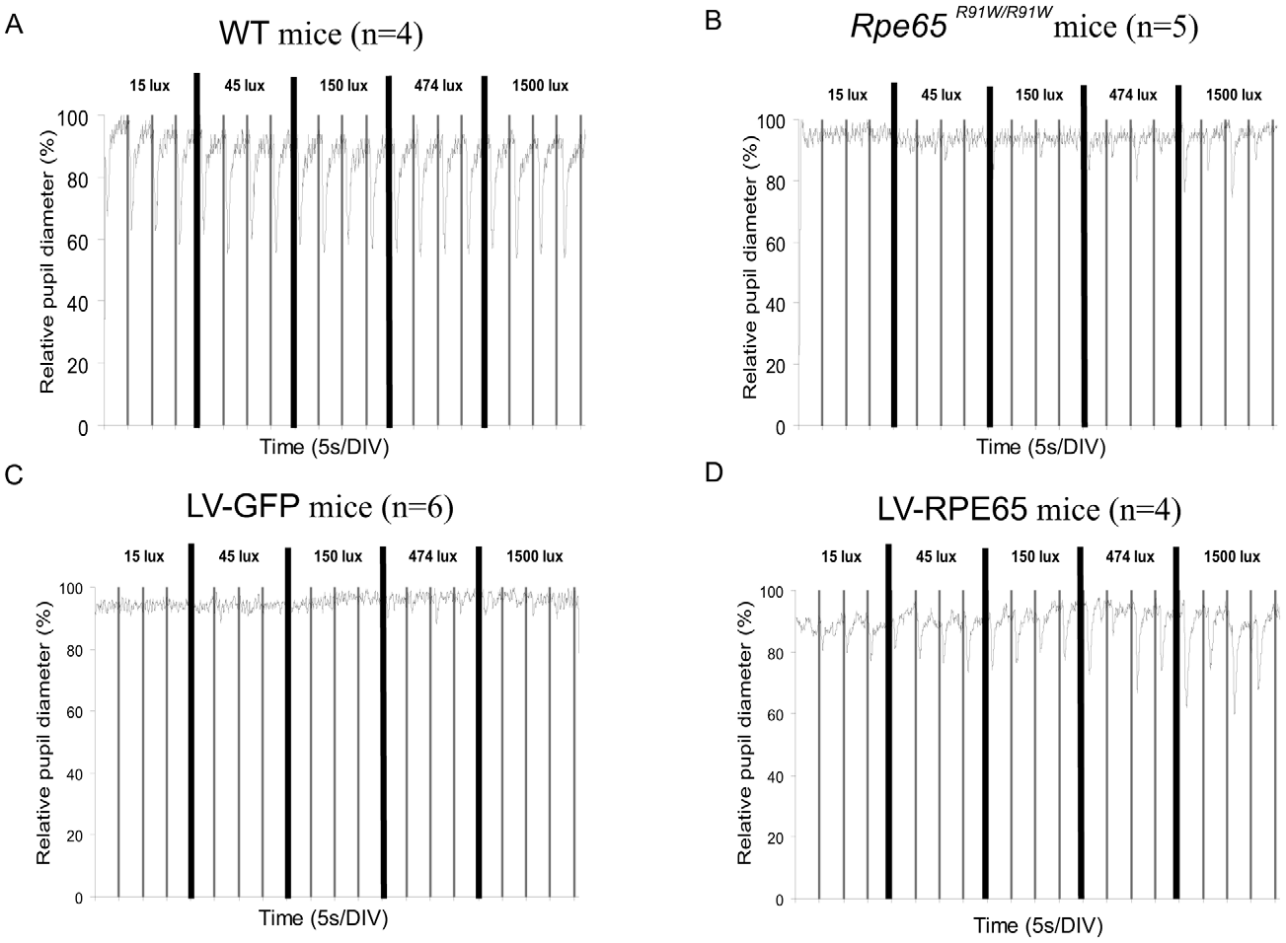
Pupillary light reflex (PLR) is improved after LV-RPE65 injection. PLR was measured following stimulation with increasing light intensities from 15 lux to 1500 lux. Each eye was independently and sequentially stimulated with 50 ms white stimuli (represented by vertical bars on the graphs) every 5 s. The pupil contraction was recorded and the diameter was plotted versus the time. (A) The average of 4 pupil responses of 1 month-old WT mice showed that eyes respond to even the lower stimulus tested without a noticeable increase of the amplitude of contraction with increasing intensity of stimuli. (B) The pupil responses from 5 eyes of 4 month-old *Rpe65^R91W/R91W^* mice were averaged and showed a severe loss of sensitivity at this age for *Rpe65^R91W/R91W^* animals. (C) The average pupil response recorded for 6 eyes injected at 1 month of age with LV-GFP shows poor pupil light reflex 3 months post injection. (D) The average pupil response recorded for 4 eyes injected at 1 month of age with LV-RPE65 shows improvement of the pupil light reflex 3 months post injection. Even for the lowest stimuli applied (15 lux), a pupil contraction is evident.

Retinal function mediates visual perception but also other vision-linked reflexes such as the pupillary light reflex (PLR). Two components are now recognized to mediate the input for PLR: a subpopulation of retinal ganglion cells (ipRGCs) and the photoreceptors. While ipRGCs are responsible for the long-lasting PLR, photoreceptors are the source for a PLR mediated by short stimuli [41], [42]. We thus recorded the PLR induced by photoreceptors in untreated *Rpe65^R91W/R91W^* mice, LV-RPE65 or LV-GFP mice treated at 1 month of age using short stimuli to emphasize on photoreceptor-dependent PLR. Each eye was stimulated alternatively while both were recorded. PLR was strongly diminished in untreated *Rpe65^R91W/R91W^* mice of 4 months of age, where only high intensity stimuli (more than 150 lux) provoked a partial contraction (Fig 5A, B). The LV-GFP group behaved similarly to untreated mice (Fig 5C). Interestingly, LV-RPE65-treated mice displayed an increase in sensitivity and PLR response was observed with the lowest stimulus tested (from 15 lux), being around 100-fold more sensitive compared to LV-GFP control groups (Fig 5D). Thus gene transfer in *Rpe65^R91W/R91W^* improves sensitivity of the PLR in concordance with the improved ERG sensitivity.

**Figure 6:**
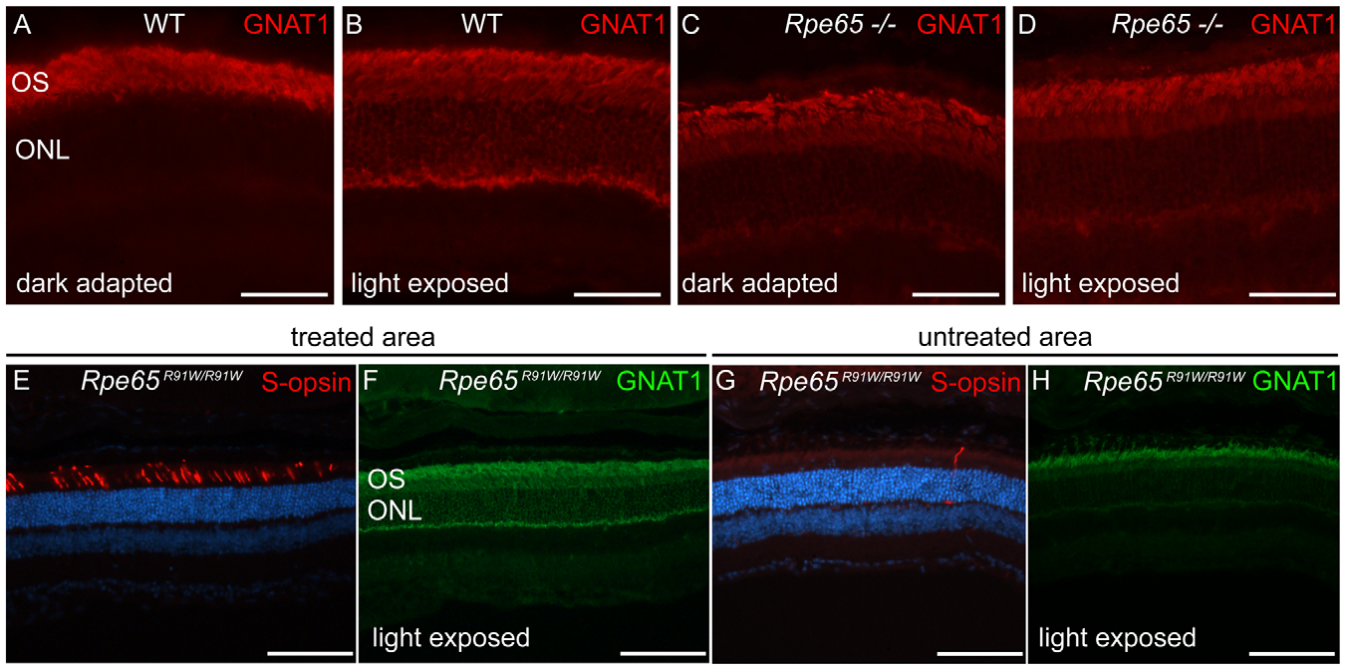
LV-RPE65 gene transfer restores light-dependent rod transducin trafficking. (A) Dark-adapted wild type mice have a high concentration of rod transducin (GNAT1, red) in the outer segments. (B) Upon light exposure (2500 lux for 20 min), GNAT1 (red) is translocated to the rod photoreceptor cell bodies. (C) In dark-adapted *Rpe65^-/-^* retina, GNAT1 labeling is also concentrated in the outer segments. (D) After light exposure, translocation of GNAT1 in rod cell bodies is strongly decreased because lack of 11-cis-retinal in these mice severely impairs phototransduction. (E,F) In *Rpe65^R91W/R91W^* mice treated with LV-RPE65 at 1 month of age, in the region of RPE65 expression easily recognizable by the correct S-opsin localization in the cone outer segments (E, red), light exposure induced GNAT1 translocation (F, green). (G,H) However in regions devoid of RPE65 gene transfer as shown by S-opsin absence or mislocalization (H, red), GNAT1 labeling remains concentrated in rod outer segments (G, green). Scale bar A to D: 50 µm; E to H: 100 µm.

In order to demonstrate an improvement of the supply of 11-*cis* retinal to the retina in the region of wt RPE65 gene transfer, we examined whether the trafficking of rod transducin α subunit (GNAT1) became light-dependent again. In the dark (Fig 6A) or in the case of absence of rhodopsin signaling in *Rpe65^-/-^* mice (Fig 6C, D), most of the protein was localized in the outer segment. However after light exposure (2500 lux for 20 min) and activation of the transduction cascade by excited rhodopsin, the GNAT1 protein was translocated to the rod photoreceptor cell bodies and feet in WT animals (Fig 6B, [43]), but not in the Rpe65-/- mice (Fig 6D). As expected, in the region protected by LV-RPE65-mediated gene transfer that was easily recognizable with corrected S-opsin localization (Fig 6E), there was a clear expression of GNAT1 all along the rods after light stimulation (Fig 6F). In contrast, GNAT1 labeling in the region of the retina devoid of wt RPE65 expression (Fig 6G) was mainly restricted to the outer segments (Fig 6H). Thus, GNAT1 labeling indirectly indicates restoration of the visual cycle in the region of wt RPE65 gene transfer.

Similarly to our previous study [13], we attempted to demonstrate cone function rescue after LV-RPE65 gene transfer. We thus injected at 1 month of age both *Rpe65^R91W/R91W^ Rho^-/-^* mice and *Rpe65^R91W/R91W^ Gnat1^-/-^* mice which are devoid of rod function, to isolate a potential cone functional rescue. No convincing cone function could be recorded 3 weeks post injection for the former or 1 and 3 months post injection for the latter mouse model despite the preserved retinal structure of the *Rpe65^R91W/R91W^ Gnat1^-/-^* mice for several months (data not shown).

### LV-RPE65 treatment in adult *Rpe65^R91W/R91W^* mice rehabilitated cones in the transduced region

**Figure 7:**
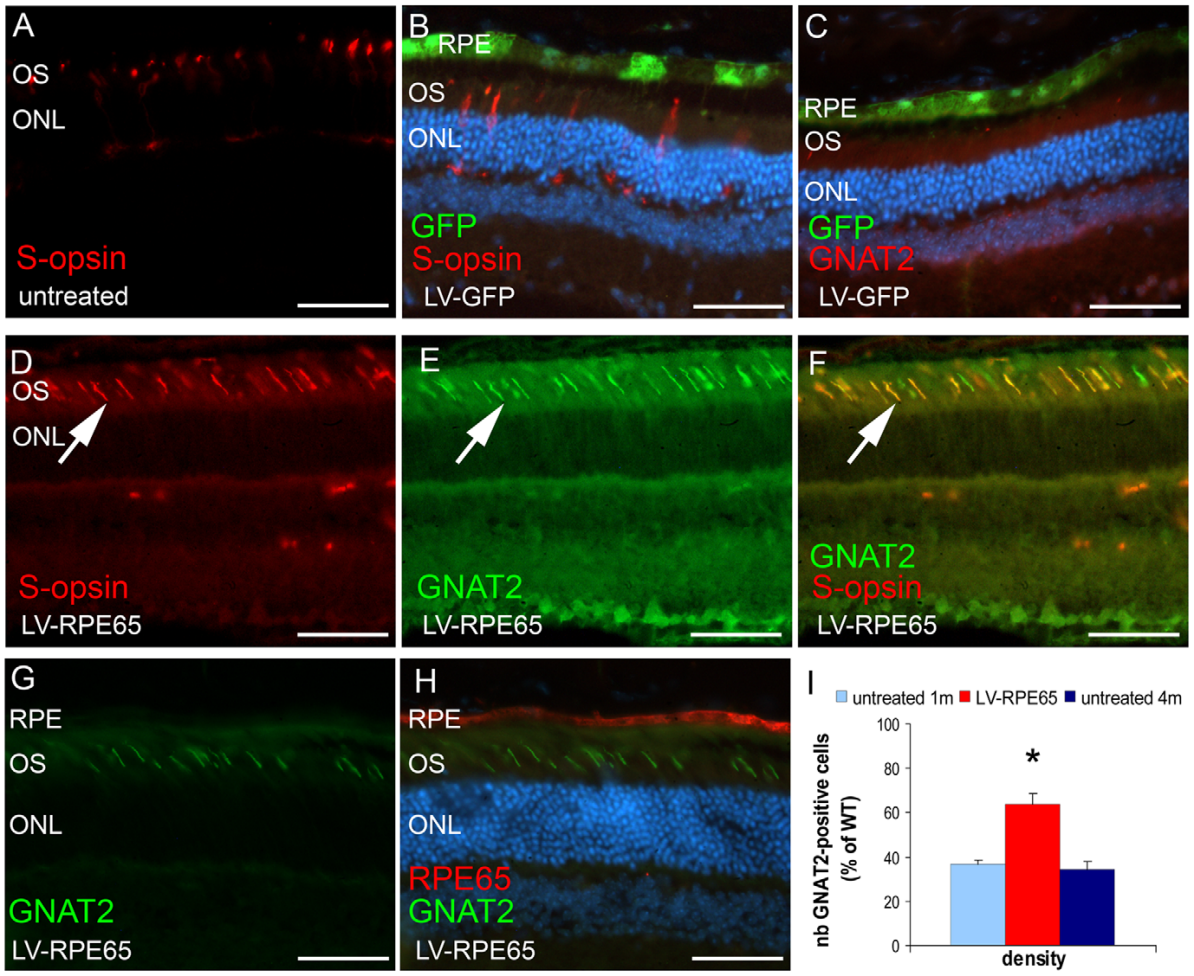
LV-RPE65 injection in 1 month-old *Rpe65^R91W/R91W^* mice protects 64% of the cones. (A,B) At 4 months of age in untreated (A) and LV-GFP- treated mice (B), residual S-opsin is seen in shorter outer segments and in cone's cell bodies and feet. (C) GNAT2 staining is rare or even absent in LV-GFP-treated mice on the ventral hemisphere. (D–H) Immunolabeling 3 months post injection of LV-RPE65 showed correct S-opsin (D,F: arrow, red) and GNAT2 (E,G: arrow, green) labeling in cone outer segments in the region of RPE65 expression (H: red). Pictures (D) to (F) were taken at a topological position in the eye similar to (A). Note in (F) that all S-opsin labeled outer segments are also GNAT2-positive (arrow, yellow). (I) Quantification using the GNAT2 staining of cone density in the middle position of RPE65 expression area showed that gene transfer preserved 64% of the cones compared to wild type. This rescue was significantly different from the value obtained from age-matched untreated *Rpe65^R91W/R91W^* mice and was also significantly higher than the value obtained with *Rpe65^R91W/R91W^* mice of 1 month of age (36% of the wt) corresponding to the time of intervention (p<0.001, star). Thus, LV-RPE65 treatment at 1 month regenerates 28% more cones that were already lacking the GNAT2 marker at 1 month of age. Data are represented as mean ± standard error of the mean (SEM). D is a merge picture of B and C; scale bar in A–F: 50 µm.

Animals were treated at 1 month of age and cone survival was then assessed three months later by immunohistochemical analysis of cone markers S-opsin and GNAT2. We observed that this relatively late delivery of wild type RPE65 protein still allowed the preservation of the expression as well as the correct localisation of S-opsin and GNAT2 (Fig 7D–H). Quantification of S-opsin-expressing outer segments in the entire section located in the center of the transduced area showed no differences between LV-RPE65 (12±2% of wild type) and LV-GFP (12±3% of wild type) or untreated groups (15±1% of wild type) despite the clear topological correlation of the correct S-opsin labeling in outer segments with the LV-RPE65 transduced area (Fig 7D, F). To note, injections in adults mainly lead to transduction of a region located in the dorsal hemisphere which is known to be under-represented by S-cones. However, similar quantification of GNAT2 labeled outer segments revealed a significant increase in the LV-RPE65 group (19±3% of wild type) compared to the LV-GFP (7±1% of wild type) or untreated groups (13±1% of wild type) (p<0.01). As these quantifications ignore the size of the transduced area which directly affects the absolute number of positive cells per section, we quantified the density of cones expressing correctly the GNAT2 marker in the region of wt RPE65 expression. With this method, the success of the rescue corresponded to 64% of the wild type density (Fig 7I). Even if this latter result showed not a full rescue compared to P5 treatment (Fig 3C), the effect of gene transfer at this time point reflects more than only preservation of cones expressing GNAT2 at the time of treatment. Indeed, the GNAT2 cone density of 1 month-old untreated *Rpe65^R91W/R91W^* mice (age of the treatment) is 36% of wild type (Fig 7I), thus treatment at 1 month restored GNAT2 correct expression in 28% additional cones that regenerated after *Rpe65* gene transfer. Moreover, in the region of wt RPE65 expression, S-opsin-positive outer segments were also GNAT2-positive (Fig 7D–F) showing that the expression patterns of both markers were corrected in the rescued cells. Co-expression of these markers strongly suggests the restoration of cone ability to respond to light.

## Discussion

The major finding of this study is that RPE65 gene transfer, and a concomitant 11-*cis*-retinal supply, allowed revitalization of cones that had lost their expression of phototransduction proteins and showed an altered expression of cone outer segment markers. The conclusion is the indication of a specific cell state during the degeneration course that can be targeted for rehabilitation toward a healthy cell state. The consequences of this observation are important for the clinical application aspect of the treatments targeting cone cells. First these results can be directly translated to gene therapy in RPE65 patients bearing the R91W mutation. Second, this study contributes to the general understanding of cone degeneration following 11-cis deprivation that occurs in several diseases due to chromophore deficiency. Both outcomes are discussed below.

The proof of principle of RPE65 gene replacement was previously clearly established in RPE65-null animals and has already lead to clinical trials [2]–[5]. However the types of mutation encountered in patients are heterogeneous and are often missense mutations [22]. Residual RPE65 activity may account for the clinical variability observed between the phenotypes [23]–[26] and could have a consequence on gene transfer efficiency. First, after gene transfer in a missense mutation background, the wild type protein (derived from the transgene) has to compete with the mutated forms, which are in place since early development, in order to achieve its function. Depending on the residual properties of the mutated proteins, interactions with partners, or the availability of the substrate may differ from natural heterozygosity, where both protein types cohabit since the beginning of their expression. This hypothesis is highly unlikely with the RPE65 R91W mutant due to its extremely low level of expression compared to the wild type protein (Fig S1C, [27]). Cell responsiveness to a therapeutic intervention may also be altered by a context of long-term mutant homozygosity. Second, the natural course of cone degeneration is different between patients and has to be assessed as precisely as possible to establish the optimal intervention time. These concerns were approached using a lentiviral-mediated RPE65 gene transfer into the *Rpe65^R91W/R91W^* mice which are homozygous for a mutation encountered in patients suffering from early-onset retinal degeneration [27].

We demonstrated that, consistently with the results obtained with the *Rpe65^-/-^* mice [13], early injection of the therapeutic vector (at P5) improves visual function and supports cone survival to a wild type level by 4 months of age. We confirmed that treatment at 1 month improves scotopic ERG sensitivity as well. This effect is coherent with the improvement of scotopic ERG observed after gene transfer in adult *Rpe65-*deficient mice which are more severely affected than *Rpe65^R91W/R91W^* mice [9], [11], [13]. Interestingly, we also found evidence of an improvement in the photoreceptor-mediated PLR, indicating that the brain visual pathway implicated in PLR is at least able to drive pupil response and can be recruited after gene transfer at 1 month of age. This observation is consistent with previous studies showing residual activity of the visual pathway implicated in PLR [44], [45], in cortical projections [46], [47]or in the optomotor reflex [48] even in the more severely affected RPE65 null background. The presence of the R91W mutated protein does therefore not impair retina functional improvement following RPE65 gene transfer.

Evidence that the visual cycle was restored after *Rpe65* gene transfer also comes from our immunohistological studies. First, light exposure induces translocation of GNAT1 from the outer segments to the rod cell bodies as a mechanism of light adaptation [43], [49]. This indicates that photons has been captured and induced the phototransduction processes. This phenomenon is only possible if the phototransduction cascade is initiated by a receptive rhodopsin protein containing the 11-*cis*-retinal chromophore. Thus, as expected in the region of increased 11-*cis*-retinal production due to gene transfer, a clear translocation of GNAT1 following light exposure is observed in rods (Fig 6). Secondly, mislocalization of the S-opsin protein was demonstrated to be a consequence of 11-*cis*-retinal deprivation and to lead to cone degeneration [27], [30], [31]. Consistent with the strong decrease in the level of 11-*cis*-retinal, *Rpe65^R91W/R91W^* mice suffer from S-opsin mislocalization at 1 month of age with a concomitant decrease in the number of S-opsin-positive cells with time [28]. At 4 months of age, the endpoint of our experiment, the number of S-opsin-positive cones corresponded to only 16% of the wild type level (personal data, [28]). After treatment either at P5 or at 1 month, in the transduced area, S-opsin was re-localized to the outer segments confirming that gene transfer induced sufficient production of 11-*cis*-retinal to correct the trafficking of the S-opsin protein. These observations show that, the increased production of 11-*cis*-retinal due to the successful RPE65 gene transfer had an impact on both rod and cone visual cells.

In 1 month-old *Rpe65^R91W/R91W^* mice 36% of the wild type cones still expressed the GNAT2 cone-specific protein. Interestingly, when animals were treated at this time point, *Rpe65* gene transfer reduced cone loss and allowed a cone preservation of up to 64% of the wild type level at 4 months of age. Such rescue was not observed in *Rpe65^-/-^* mice (where only 3% of the cones still express the GNAT2 marker at 1 month of age, unpublished data) demonstrating a prolonged therapeutic window in the *Rpe65^R91W/R91W^* model. The success of cone rescue after gene transfer at 1 month in *Rpe65^R91W/R91W^* mice is a direct consequence of the slower cone degeneration observed in these mice and may have a major impact for a human application. Indeed, this knock-in mouse model is more similar to the patients bearing missense mutations than the *Rpe65^-/-^* mouse model [27]. These patients may thus also benefit from a longer therapeutic window compared to patients suffering from null mutations.

The second main advance illustrated by the success of treatment at 1 month in *Rpe65^R91W/R91W^* mice is that not only did the intervention stop cone loss and maintain the 36% of GNAT2-expressing cones present at this age, but 28% additional cones were also recruited to correctly re-express the GNAT2 cone transducin protein, or at least to restore a detectable level of the protein. Indeed the ventral expression of the S-opsin protein is severely impaired in the retinas of 1 month-old *Rpe65^R91W/R91W^* mice while the expression of the GNAT2 protein is drastically reduced both dorsally and ventrally (36% of the wild type level). Quantitative PCR showed that S-opsin transcription is already reduced to 43% at this age and fell to around 10% at 4 months of age, while GNAT2 transcription slowly decreased from 80% to 40% [28]. Thus, while the S-opsin mRNA level severely drops, GNAT2 is primarily affected at the protein level. The necessity of light capture ability and preserved phototransduction for photoreceptor survival is now widely accepted. Not only do mutations of phototransduction proteins lead to retinal degeneration [50] but they also impact on the compartimentalization of other proteins [31], [51]–[53]. Our work supports the notion that, similarly, the 11-*cis*-retinal supply is essential for correct expression and localization of cone proteins. Once the cone opsins are correctly expressed and can capture light, the expression of phototransduction proteins such as GNAT2 can be re-established. Another possibility would be that 11-*cis-*retinal acts as a cofactor for the transcription of specific genes. Whichever mechanism is implied, gene transfer at 1 month of age allowed to recover GNAT2 protein up to 3 months post injection in 28% additional cones that had lost the GNAT2 protein expression at the time of treatment. This rescue was associated to the normalization of S-opsin protein expression in the same cells, as well as improvement of outer segment structures, showing that these cells are rehabilitated to capture light, and suggesting that cone function is restored as well.

We previously showed that restoration of the cone marker expression correlates with the cone function state after gene transfer using *Rpe65^-/-^ Gnat1^-/-^* mice [13]. Unfortunately we were unable to show a rescue of cone function so far, using either the *Rpe65^R91W/R91W^ Rho^-/-^* mice injected at P5 or 1 month of age, or the *Rpe65^R91W/R91W^ Gnat1^-/-^* model injected at 1 month of age. The rod degeneration induced in the *Rho^-/-^* background may interfere with a potential cone rescue. However, the lack of cone function recovery after P5 treatment contrasts with the study of Pang *et al.* who were able to recover a significant cone function in *Rpe65^-/-^ Rho^-/-^* animals 4 weeks after treatment at P14 [18]. Differences in the type of vector used (ssAAV and a strong ubiquitous promoter) or the injection procedure may explain these discrepancies. Moreover the rod function loss occuring prior to the gene transfer could also have a deleterious effect on the cone and rod environment (less neurotrophic support, release of toxic agents, etc.) in the more stable *Rpe65^R91W/R91W^ Gnat1^-/-^* model, thus preventing functional cone rescue. Another possibility is that, despite the success in increasing expression of cone markers in *Rpe65^R91W/R91W^* retinas, treatment at 1 month of age is too late to allow cone function recovery as well. Finally, as we observed a limited effect with the early treatment of *Rpe65^-/-^ Rho^-/-^* mice compared to the study of Pang *et al.* (2010) [18], we may also have to improve our protocol in order to increase the dose of RPE65 re-expression to allow a functional cone recovery in these models. Therefore, further experiments are needed to demonstrate cone function rescue after gene transfer in the *Rpe65^R91W/R91W^* mouse model. The difficulty of this model is mainly due to the residual cone activity arising from the low amount of 11-cis retinal, and to rods which, with minute amounts of chromophore, behave as cones. Both of these activities mask the beneficial effect of the gene therapy.

The importance of 11-*cis*-retinal in preserving cone survival confirms previous observations [27], [28], [30]–[32], [54]. The mislocalization of cone opsin is similar in other models deprived of 11-*cis*-retinal (*Lrat^-/-^* [30], [31], *Irbp^-/-^* [32]). However the kinetics of the cone cell loss and the shortening of outer segments probably depend on the severity of the mutations (complete or partial loss of 11-*cis*-retinal regeneration). The determination of the critical time frame, when cones are deprived of phototransduction proteins but can be re-mobilized to express them correctly (as shown by our results), is an important point in order to be able to offer visual rescue to patients. Some studies have already approached this topic notably using measurement of the retina thickness in the RPE65-affected patients as a criterion to determine the retinal degeneration stage [20]. It is here of prime importance to note that measuring the thickness of the outer nuclear layer, which reflects the number of photoreceptor cell bodies still present in the retina, using optical coherence tomography (OCT), is indeed a reliable reference that can illustrate the amount of cell loss in patients. However, the measurement of the thickness of the outer segments which are severely affected in remaining cells deprived of correct phototransduction proteins may lead to an underestimation of the potential therapeutical effect. This hypothesis could be challenged only if “ghost photoreceptors” negative for GNAT2 and for S-opsin proteins can be detected by another marker allowing to investigate the size and the potential of regeneration of the outer segments. There is no data so far on cone gene expression during the natural course of the disease in human but our data suggest that a delay between cone protein defects and cone death may also occur in the patients.

Finally our results illustrate the importance and the advantages of animal models bearing identical mutations to those encountered in human diseases for preclinical studies. In consequence, they also highlight the importance of genotyping and phenotyping the patients precisely in order to help design the appropriate models and to collect relevant information from preclinical studies. These data will subsequently offer the best conditions for translation to human applications.

## Materials and Methods

### Ethic statement

The animals were handled in accordance with the statement of the “Animals in Research Committee” of the Association for Research in Vision and Ophthalmology, and protocols were approved by the local institutional committee, the “Service de la consommation et des affaires vétérinaires du canton de Vaud” (autorisation VD#1367.3).

### Lentiviral vectors

The lentiviral vectors used in this study were previously described in Bemelmans et al. 2006 [13]. Briefly, the transgene plasmids contain the central polypurine tract and central termination sequence (cPPT/CTS, [55]), the R0.8 promoter (800 bp of the human RPE65 promoter [39], [40]) which drives expression of the mouse RPE65 cDNA (LV-RPE65 vector) or the GFP gene (LV-GFP vector), and the woodchuck hepatitis virus post-transcriptional regulatory element (WPRE, [56]) downstream of the transgene.

Recombinant lentiviral particles were produced by transient transfection of 293T cells, as previously described [38], [57]. Viral supernatants were concentrated by two successive ultracentrifugations at 70′000 g and 4°C for 90 minutes. Total particle concentration of the viral stocks was estimated by quantification of the p24 capsid protein using RETRO-TEK HIV-1 p24 Antigen ELISA kit (ZeptoMetrixCorporation, Buffalo, NY USA) according to the manufacturer's instructions. In addition, infectious titers of the LV-GFP vector were quantified by infection of 293T cells, in which the R0.8 promoter is active, followed by flow cytometry on a FACSCalibur (Becton Dickinson, Franklin Lakes, NJ, USA).

### Animals and Surgical procedures

The mice were kept at 20°C under a 12 hours light/12 hours dark cycle with light on at 7 am and fed ad libitum. The mice were anaesthetized with volatile anaesthesia, and injections at post-natal day 5 (P5) (intravitreously) or at 1 month of age (subretinally) were performed as described in Bemelmans *et al.* 2006 [13]. For P5 treatment, 1.5 µl of viral suspension containing 140 ng or 100 ng of p24 for LV-RPE65 and LV-GFP, respectively, were injected per eye, while for 1 month treatment the same dose was injected but in 2 µl of viral suspension per eye. After surgery, 0.5 mg/ml of paracetamol was added in the water of the mice for 1 day.

### Electroretinogram (ERG)

ERG recordings were performed on site at Jules-Gonin Eye Hospital as described in Bemelmans et al 2006 [13] using a Multiliner Vision apparatus (Jaeger/Toennies, Höchberg, Germany) and a Ganzfeld stimulator adapted for rodent examination. Retinal function was assessed with corneal DTL electrodes. Amplitude of the a-wave (photoreceptor response) was defined as the difference between the baseline level at the time of stimulation and the peak of the a-wave. Amplitude of the b-wave (second-order neurons) was defined as the difference between the peak of the b-wave and the peak of the a-wave (or the baseline level when the a-wave was not detectable). Amplitudes are expressed in microvolts (µV).

### Pupillary light reflex (PLR)

The A1000 pupillometer for small rodents was developed by Neuroptics with the collaboration of Dr Randy Kardon (University of Iowa) and Dr Sinisa Grozdanic (Iowa state University) (manuscript in preparation). The mice were anaesthetized with a mixture of ketamine (45 mg/kg) and xylazine (17 mg/kg). A protocol using 50 ms white light stimuli of increasing intensities (15, 45, 150, 474 and 1500 lux) with 5 s of interval was applied twice sequentially to the left and then to the right eye. The evoked pupil response was recorded by two infrared cameras targeting both pupils and the pupil diameter determined automatically by the Neuroptics software. The averages of 4 to 6 pupil recordings were then calculated and plotted using the Excel program.

### Histology and immunolabeling

Before their sacrifice at 4 months of age, the mice injected at 1 month were dilated by topic administration of 0.5% tropicamide + 10% neosynephrine (1: 1) and exposed for 20 min to 2500 lux in cages covered with aluminium foil to increase translocation of the rod transducin to the rod cell body. After sacrifice, the mouse eyes were cauterized at the nasal corner of the eye, enucleated, the corneas were perforated and eyes were fixed for 1 hour in 4% PFA in PBS. After overnight incubation in 30% sucrose, the eyes were embedded based on the cauterization mark in albumin from hen egg white (Fluka, Buchs, Switzerland) in order to provide sections with both a dorsal and a ventral hemisphere, and cut in 14 µm sections using a cryostat. Sections were collected on six serial slides for each eye allowing multiple labeling throughout the entire eye for each slide.

The antibodies against GNAT2, S-opsin, RPE65 and GFP and the immunohistological conditions were already described in Bemelmans *et al.* 2006 [13]. Rabbit anti-GNAT1 (Santa Cruz) was used at 1∶2000, overnight at 4°C. Secondary antibodies, goat anti-rabbit linked to Alexa 488 (1∶4000, Invitrogen, Carlsbad, CA, USA) or to Cy5 (1∶500, F(ab) fragment, Jackson Immunoresearch, Newmarket, England), were incubated for 1 hour at room temperature. For co-labeling of both polyclonal antibodies anti-RPE65 (Pin5) and anti-GNAT2, anti-RPE65 was first incubated overnight and well washed before incubation for 6 hours at room temperature with anti-rabbit-Cy5 F(ab) fragments to saturate the epitopes [58]. Then we proceeded with the standard protocol for GNAT2 staining.

Quantifications in the transduced areas of GNAT2 and S-opsin were performed as previously described [13]. In order to obtain the ratio to the wild type for each treated eye, we averaged GNAT2 and S-opsin countings of corresponding sections (using section of the same position in the eye on the nasal-temporal axis) of 3 to 4 different wild type eyes as control values. Densities of GNAT2-labeled outer segments were determined by the average density at a given location on a section, for 3 sections located in the center of the transduced area (most transversal sections). Densities of the identical location on 3 similarly positioned sections of wild type retinas of were averaged as controls. Similar quantifications were performed in 1 month- and 4 month-old untreated *Rpe65^R91W/R91W^* mice.

### Statistical analysis

Statistical analyses of the electroretinograms were done using StatView® 5.0 software. Histological counting of cone markers was analyzed by one way-ANOVA to determine the statistical significance between the different groups (LV-RPE65 treated, LV-GFP treated, untreated *Rpe65^R91W/R91W^*).

## Supporting Information

Figure S1 **Lentiviral-mediated** ***Rpe65*** **gene transfer in the retina.** (A) Injection of LV-RPE65 at P5 allowed expression of the wt RPE65 in the RPE (labeled in green). (B) Injection of LV-GFP occasionally led to GFP expression in Müller cells (labeled in green). (C) Subretinal injection of LV-RPE65 in 1-month old *Rpe65^R91W/R91W^* mice also induced expression of the wt RPE65 protein (labeled in red). The limit of staining between the faint R91W mutant protein detection and the strong WT RPE65 expression is indicated by an arrow. (D) The transduced area was comparable between P5 and 1 month injections. The heterogeneity observed is due to the surgical procedure. RPE: retinal pigment epithelium; OS: outer segment; ONL outer nuclear layer (photoreceptor nuclei); the scale bar indicated in A represents 50 µm for A and B and 200 µm for C.
(TIF)
