# Supplementary figures and images for: Gene Therapy Regenerates Protein Expression in Cone Photoreceptors in Rpe65R91W/R91W Mice
Source: PLoS One. 2011 Feb 3;6(2):e16588. doi: 10.1371/journal.pone.0016588 (PMC3033393; doi:10.1371/journal.pone.0016588)

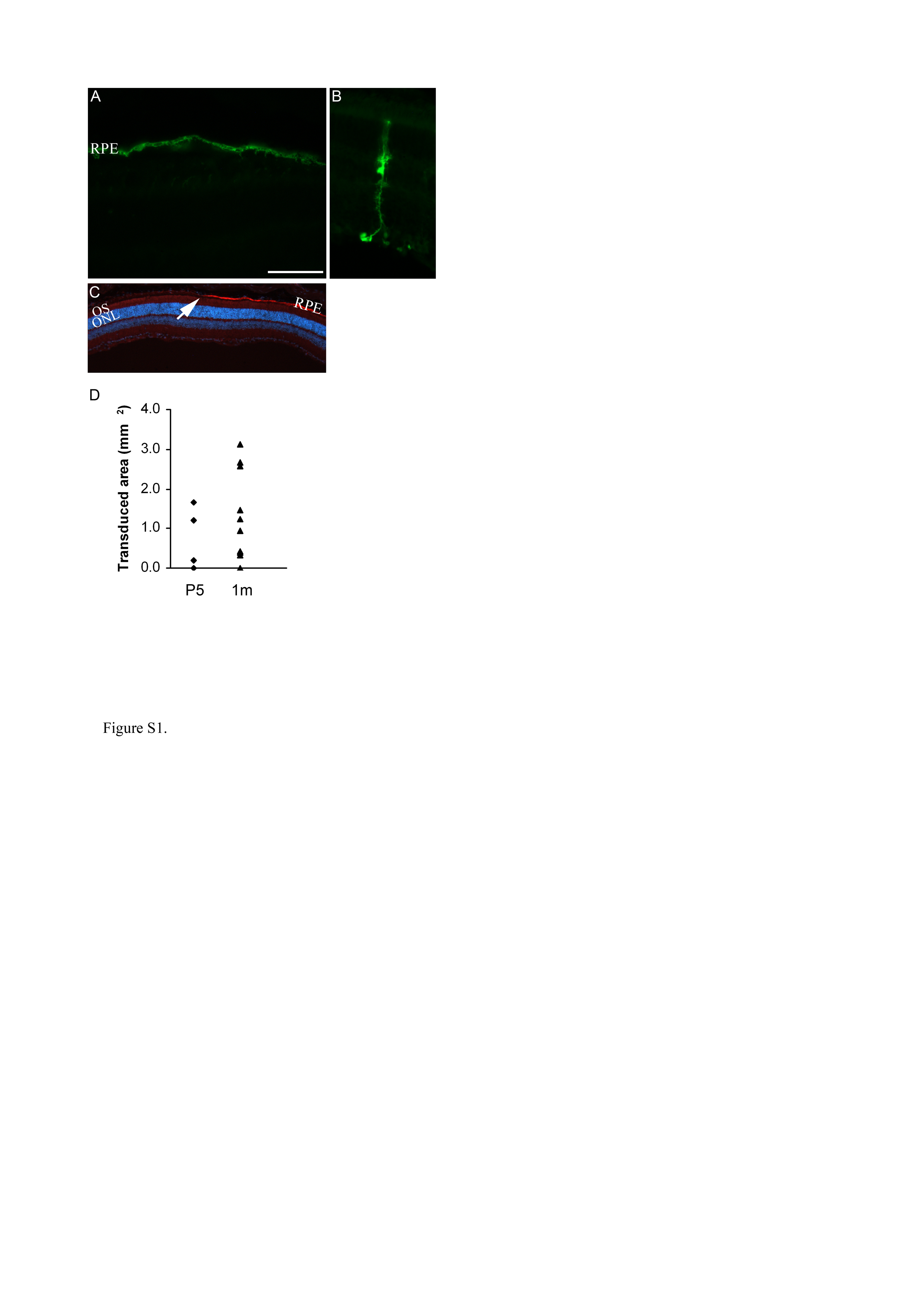

Supplement: Figure S1 — Lentiviral-mediated Rpe65 gene transfer in the retina. (A) Injection of LV-RPE65 at P5 allowed expression of the wt RPE65 in the RPE (labeled in green). (B) Injection of LV-GFP occasionally led to GFP expression in Müller cells (labeled in green). (C) Subretinal injection of LV-RPE65 in 1-month old Rpe65R91W/R91W mice also induced expression of the wt RPE65 protein (labeled in red). The limit of staining between the faint R91W mutant protein detection and the strong WT RPE65 expression is indicated by an arrow. (D) The transduced area was comparable between P5 and 1 month injections. The heterogeneity observed is due to the surgical procedure. RPE: retinal pigment epithelium; OS: outer segment; ONL outer nuclear layer (photoreceptor nuclei); the scale bar indicated in A represents 50 µm for A and B and 200 µm for C. (TIF) [file pone.0016588.s001.tif]
